# Decay radius of climate decision for solar panels in the city of Fresno, USA

**DOI:** 10.1038/s41598-021-87714-w

**Published:** 2021-04-21

**Authors:** Kelsey Barton-Henry, Leonie Wenz, Anders Levermann

**Affiliations:** 1 Potsdam Institute for Climate Impact Research, Potsdam, Germany; 2 Institute of Physics, Potsdam University, Potsdam, Germany; 3 Mercator Research Institute On Global Commons and Climate Change, Berlin, Germany; 4 Department of Agriculture and Resource Economics, University of California, Berkeley, USA; 5 Columbia University, New York, NY USA

**Keywords:** Statistics, Climate-change policy, Environmental economics, Psychology and behaviour, Sustainability, Climate change, Climate-change mitigation, Computational science

## Abstract

To design incentives towards achieving climate mitigation targets, it is important to understand the mechanisms that affect individual climate decisions such as solar panel installation. It has been shown that peer effects are important in determining the uptake and spread of household photovoltaic installations. Due to coarse geographical data, it remains unclear whether this effect is generated through geographical proximity or within groups exhibiting similar characteristics. Here we show that geographical proximity is the most important predictor of solar panel implementation, and that peer effects diminish with distance. Using satellite imagery, we build a unique geo-located dataset for the city of Fresno to specify the importance of small distances. Employing machine learning techniques, we find the density of solar panels within the shortest measured radius of an address is the most important factor in determining the likelihood of that address having a solar panel. The importance of geographical proximity decreases with distance following an exponential curve with a decay radius of 210 meters. The dependence is slightly more pronounced in low-income groups. These findings support the model of distance-related social diffusion, and suggest priority should be given to seeding panels in areas where few exist.

## Introduction

In recent years, the growth of residential solar photovoltaic power generation systems and programs to spur their implementation has led to both increased data on installation patterns and study of the dynamics of their uptake. Peer effects on climate-related decisions have been identified through both passive, geographic effects (e.g. I see a panel close to my house) and active, social network effects (e.g. I hear about panels via word of mouth, or have similar tendencies to others with my socio-demographic or educational background) have both been suggested as important motivators for installation^1^. Distinguishing between these types of effects is particularly difficult given that previous studies have been conducted on highly aggregated spatial levels (ZIP codes or census tracts), and peer effects may be confounded within these areas by the existence of similar trends within neighbourhoods that share social, demographic, or economic qualities. Research on a finer geographic resolution will allow a better understanding of the distance sufficient to induce a potential passive peer effect. We seek to answer these questions jointly: what is the more effective type of peer effect (passive or active), and what potential effect does distance have on this passive mechanism.

Closer physical proximity to solar panels has been identified as having a positive effect on implementation over a zip-code or district, and across spaces larger than 1km^2–5^. It has also been shown that highly localized diffusion is most influential on the likelihood of implementation, but less is known about diffusion dynamics within 1 km^3,6^. Previous qualitative research in Sweden has suggested that simply viewing, or living in proximity to a solar panel are of negligible influence on the likelihood of others installing panels, and that peer effects are generated through existing social networks^7,8^.

Both passive and active effects have been identified as having significant effects on the uptake of alternative fuel vehicles and resource conservation behaviors^9,10^. Mildenberger et al. provide support for the active channel, finding that high levels of political activity are an important predictor of solar panel installation, regardless of political affiliation^11^. Identifying those mechanisms through which climate decisions, such as solar panel installation or uptake of alternative fuel vehicles, are made, has the potential to support pro-climate decision making and help governments design effective policies at potentially lower cost.

**Figure 1:**
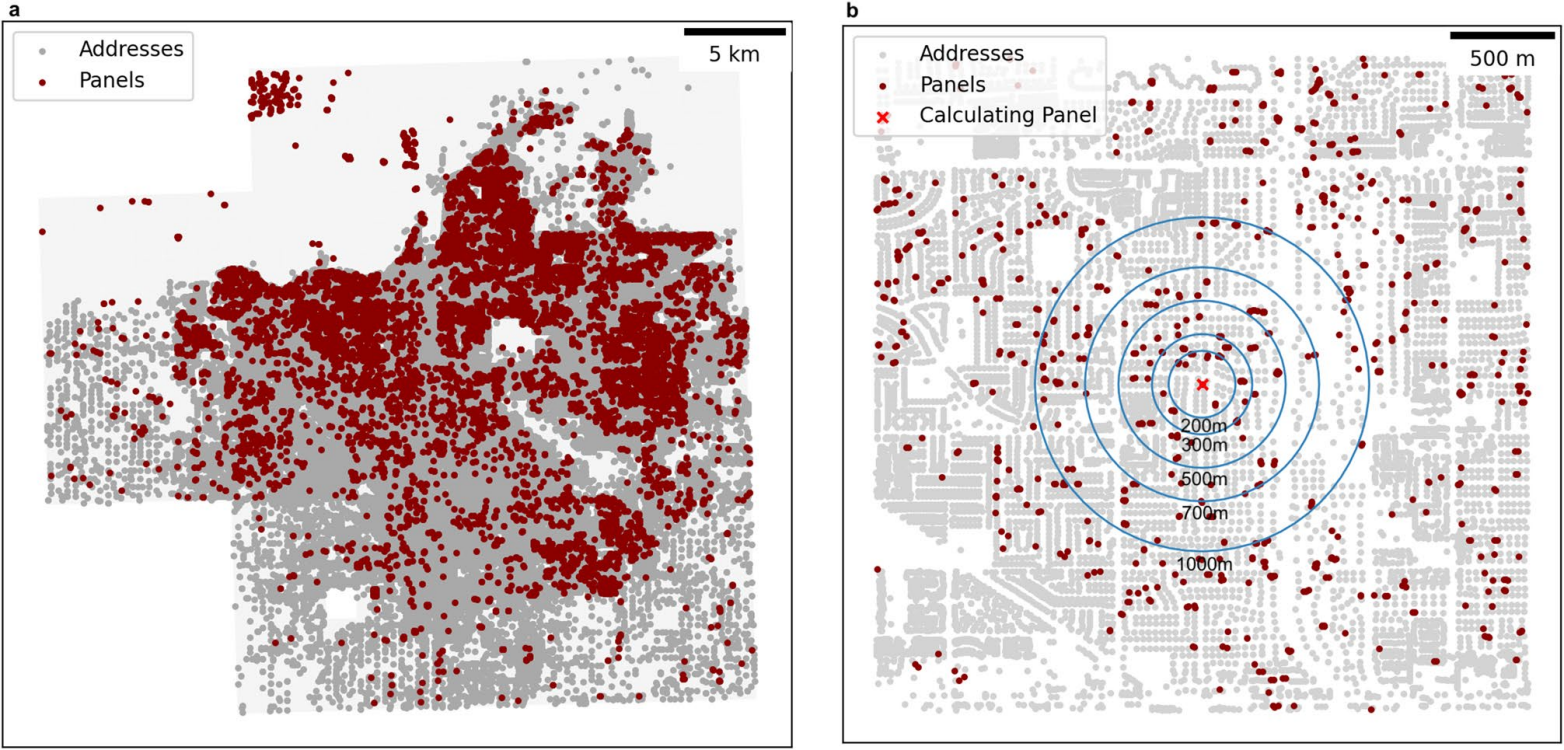
**Geolocations of solar panels and addresses in Fresno included in the analysis**. In panel (a), light grey boxes indicate bounds of the aerial images in which the solar panel geolocations are marked. Each address is marked with a grey dot, and each panel geolocation is marked by a red dot. Geolocations of solar panels and bounding boxes of the aerial images these data were derived from are taken from Bradbury, et al. (2016), address data for the city of Fresno is made publicly available by the County of Fresno^13^. Panel (b) is a close-up illustrating the panel density calculation process, the red ‘x’ indicates the panel around which panel density is calculated, with several example radii provided in blue.

Here, we aim to shed light on the mechanisms through which this peer effect occurs by analysing solar panel uptake at the level of actual households. This is made possible by a data set of geo-located solar panels for the city of Fresno, identified from satellite images^12^ (Fig. 1a). We map these data with geo-located address and school district data provided by the County of Fresno^13^ and Institute for Education Science^14^ as well as with socio-economic and demographic variables on the census tract level from the American Community Survey^15^ (see “Data and methods” section for detail).

We employ feature importance analysis in conjunction with machine learning techniques to identify the most important predictor for solar panel installation, taking panel density within varying radii ranging from 200 m to 1200 m around a house into account. This enables identifying the effect of proximity over space as well as any decline in effect size within this distance. We also are able to examine the effect of proximity to a solar panel, regardless of the type of building on which it is installed. Given that our data includes both proximity to a panel and a potential social network (school district), we are able to compare the potential effects on uptake through both of these mechanisms.

We employ tree-based machine learning techniques because they do not specify the functional form of the relationships between features and the outcome, and are therefore able to capture nonlinear and non-parameterized relationships. Additionally, tree algorithms also demonstrate better performance on non-linearly separable or highly correlated data compared to parameterized modelling methods^16,17^. This is especially important given the nature of solar panel installation data, in which the outcome classes (having a panel or not having a panel) are highly unbalanced: homes with installed panels are far fewer than homes without. This problem can be so extreme that examples of installed photovoltaic systems can apply to less than 1% of the total buildings in the data^4^. Machine learning methodologies, especially tree-based algorithms, have been found to be better able to handle these types of imbalanced class problems and consistently show better performance than statistical modelling techniques^16,18^.

### Panel density most important predictor for solar panel implementation

Specifically, we train an AdaBoost classifier to predict the likelihood of a particular address having a solar panel based on the socio-economic, demographic, and solar panel density features associated with that address (an overview of all features included in the main model can be found in Supplementary Table S1 and a discussion of all classifiers and evaluation metrics considered in the “Data and methods” section). We consider densities ranging from 200 m to 1200 m (in 100 m increments) and construct a different model for each density measurement, to avoid introducing high collinearity between the density variables constructed at different radii. Panel density is normalized by address density. Both the density of panels around a particular address and the density of addresses around that address are calculated exclusive of that address and its possible panels. Excluding an address’ own panel and calculating the density using just those other panels within the chosen radius ensures we do not induce a data leakage problem. A visualization of the radii constructed around a panel is included in Fig. 1b.

We then examine the contribution of each feature to the accurate prediction of an address’ solar panel status (having or not having a panel) by calculating the permuted importance of each feature. That is, we compare the importance of each feature to others in the model based on their contribution to the model’s performance (performance defined here by the area under the precision-recall curve, AU P-R Curve; see “Data and methods” section for detail). As the feature importance scores do not describe the direction of influence of that variable on the outcome (positive or negative), we check this direction by calculating the correlation between all variables and the outcome for all models. In addition to our primary evaluation metric, AU P-R curve, we also determine the total accuracy, the receiver operating characteristic curve (AUC ROC) as well as the confusion matrices for all classifiers (Tables S2-S4, Figures S1-S6; see Supplementary Discussion 1, and “Data and methods” section for a detailed description). Based on these metrics, we determine our preferred model.

**Figure 2:**
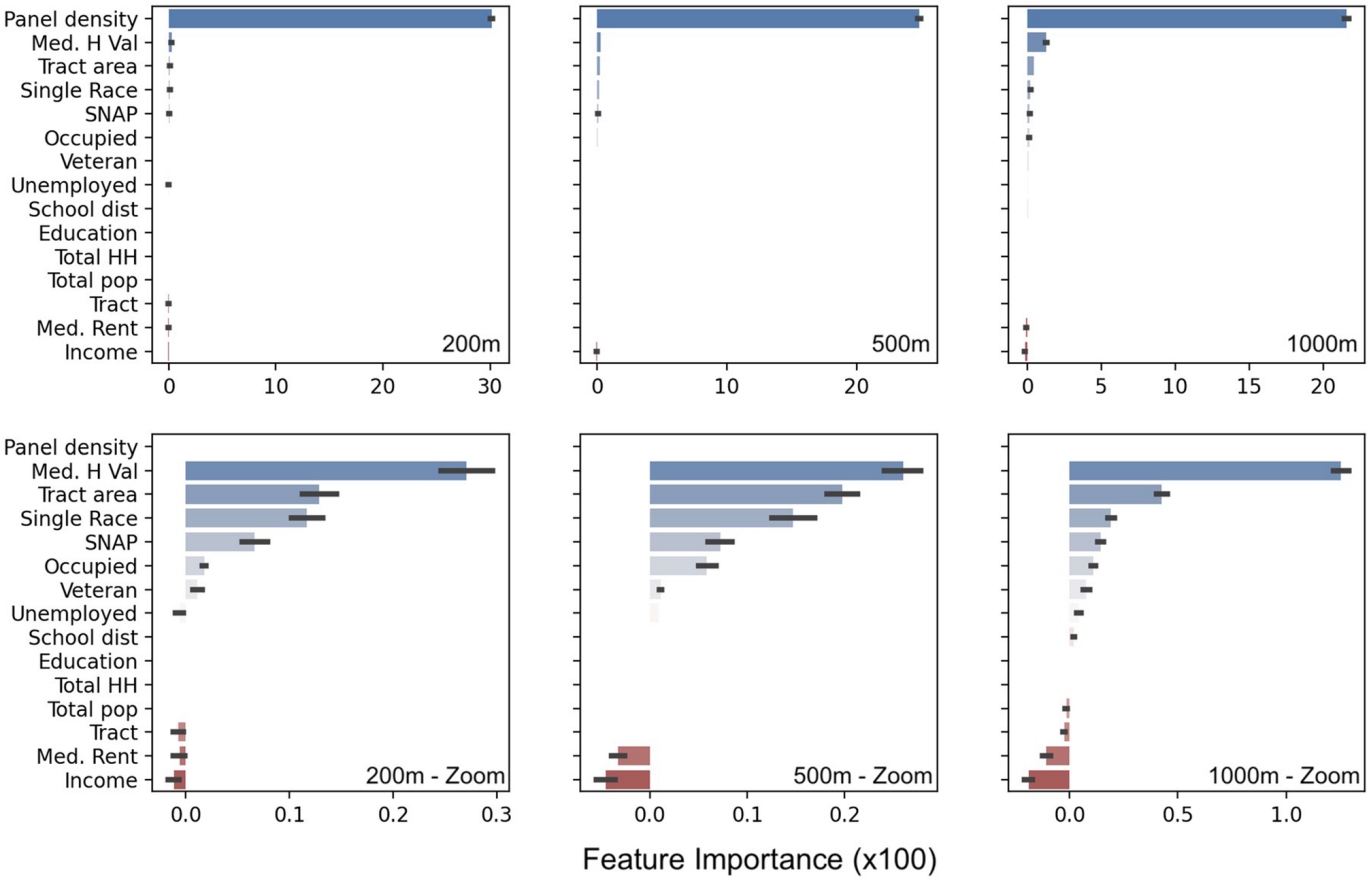
**Panel density most important feature for predicting solar panel installation**. Feature importance scores by variable are shown as point value of performance contributed by each feature for all features, multiplied by 100. The solar panel densities shown are calculated at 200 m, 500 m, and 1 km around each address in the dataset (see Supplementary Table S5 for results for all 100 m increments between 200 m and 1200 m). The top panel shows the bar plot of all features in the model, the lower panel shows a zoomed view of all features excluding the density feature for each model. Across all models, panel density consistently contributes the largest gains in performance. Feature importance is highest for a radius of 200 m and decreases with radius length.

In all models, normalized panel density at a particular address is the most important feature by a large margin, and with a calculated p-value significant at the 1% level (Supplementary Table S5). It surpasses all economic (e.g. median household income, employment status), housing (e.g. home value, owner occupied homes), demographic (e.g. racial breakdown, median age), and network (school district) variables (Fig. 2). These results are robust for all radii at which density is calculated with the feature increasing in importance as the radius is shortened. Secondarily important variables include median household income and median home value, as can be seen in the close-up panels of Fig. 2. In all models, we find that the normalized density is positively correlated with our outcome, confirming that solar panel density around an address is a positive predictor of an address having a solar panel installed (see “Data and methods” section for more detail**)**. Model performance suffers across all models when density variables are removed (Supplementary Discussion 2; Supplementary Table S6 and Supplementary Figure S7). This further underlines the predictive power of the panel density variable to predicting the presence of a solar panel at a particular address. Applying a simple regression model further supports our main conclusion about the influence of proximal solar panel density on the likelihood of installing a panel, and its positive influence (Supplementary Tables S7-S9). Panel density also remains the most important variable in all models when averaged over census tract (Supplementary Figures S8 and S9).

### Exponential decay of panel density importance with larger radii

Comparing importance scores of density variables calculated at different radii across these radii revealed a larger influence of panels within shorter distances. To further explore how the importance of panel density decreases with distance, we calculate in a next step the normalized density of panels to addresses at each radius with the panels and addresses from the previous radius subtracted. Feature importances for density variables calculated without the subtraction of the previous radius are provided as robustness check and show qualitatively the same result (Supplementary Figure S10). The subtraction of the previous radius’ density isolates the effect of just the area of increase in radius from the previous model, containing a shorter radius. The same set of socio-economic and demographic variables are included in all models, regardless of the radius over which density was calculated.

**Figure 3:**
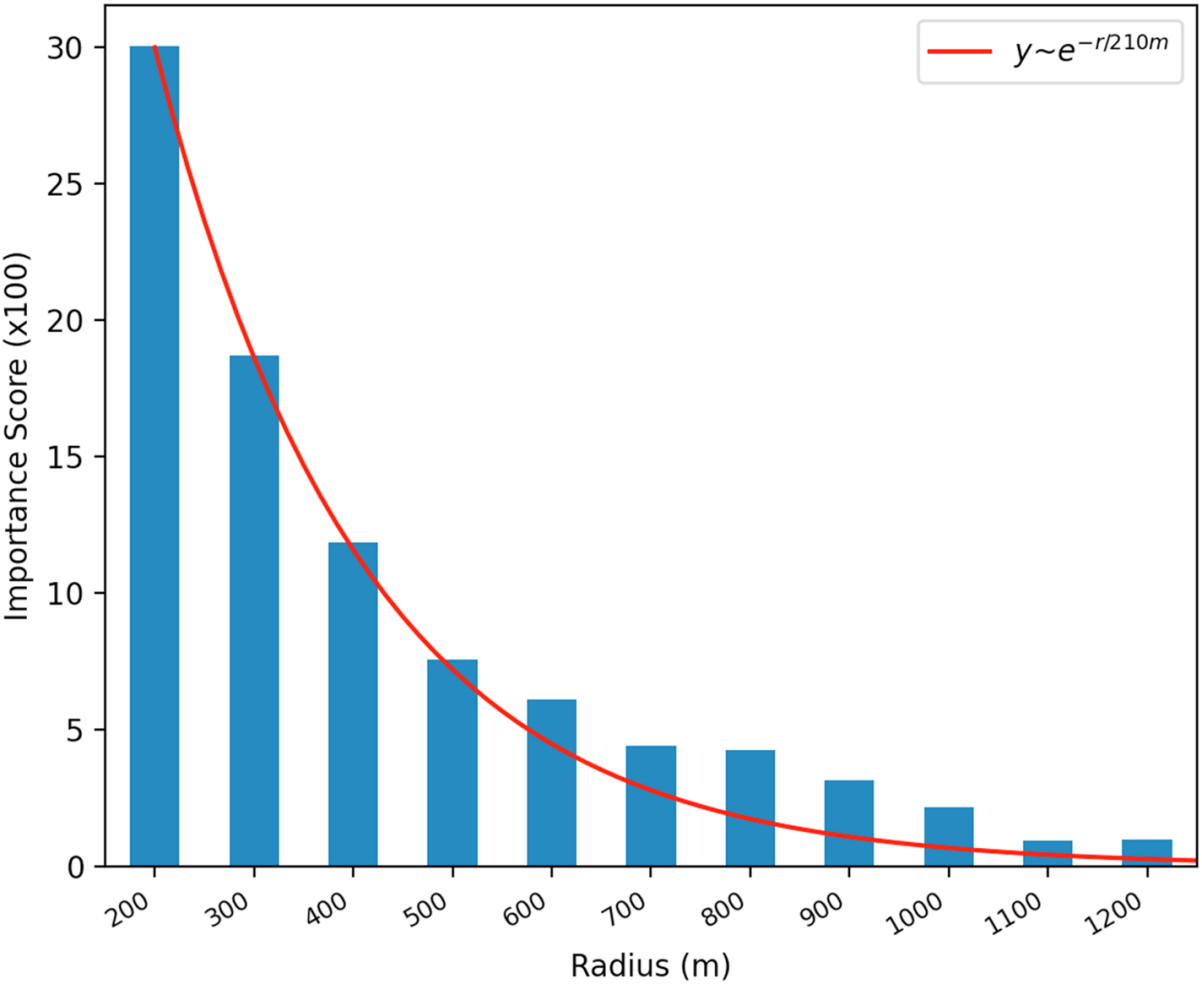
**Exponential decay of panel density importance with larger radii.** Feature importances (presented multiplied by 100) are derived from 10 different models, each with the same set of socio-economic and demographic variables. In each model, a different radius is chosen over which density is calculated where just the increase in area compared to the next smaller radius is considered. The height of the bar indicates the improvement in model performance due to the inclusion of that density feature. The data follow an exponential curve with a decay radius of 210 m.

We find that the density of panels around an address becomes a less important feature in the model as the radius over which we calculate this density grows (Fig. 3). The panel density variable with the smallest radius (200 m) has the largest contribution to model performance of all panel density features. The data follow an exponential decay with a radius of 210 m. This indicates that those buildings located closest to an address most strongly predict the likelihood of an address also having a solar panel, with this peer effect decreasing exponentially as distance from the address increases.

This relationship persists when the data are grouped either by tract area or by number of households into three bins (Supplementary Figures S11 and S13). Even though sub-selecting data reduces the clarity of the signal and therefore the performance of the classifier, we find the exponential decay function with a radius of 210 m is still a very good fit for these sub-groups in both analyses (Supplementary Figures S12 and S14). This suggests that the decay effect we see is not motivated by a correlation to tract size either by physical size or number of households, but rather a true effect of proximity.

As the radius over which density is calculated surpasses 500 m, there is a stagnation in the predictive power of the panel density feature, and it ceases to be a highly important feature, demonstrated by the decrease in the feature importance score. P-values indicate that all panel density variables across all models remain significant at a 1% level (Supplementary Table S10). These results indicate that there may be a nonlinear relationship between proximity to solar panels and the likelihood of a panel being installed at a near address, which may be bounded around 500 m.

### Importance of solar panel density moderated by income

**Figure 4:**
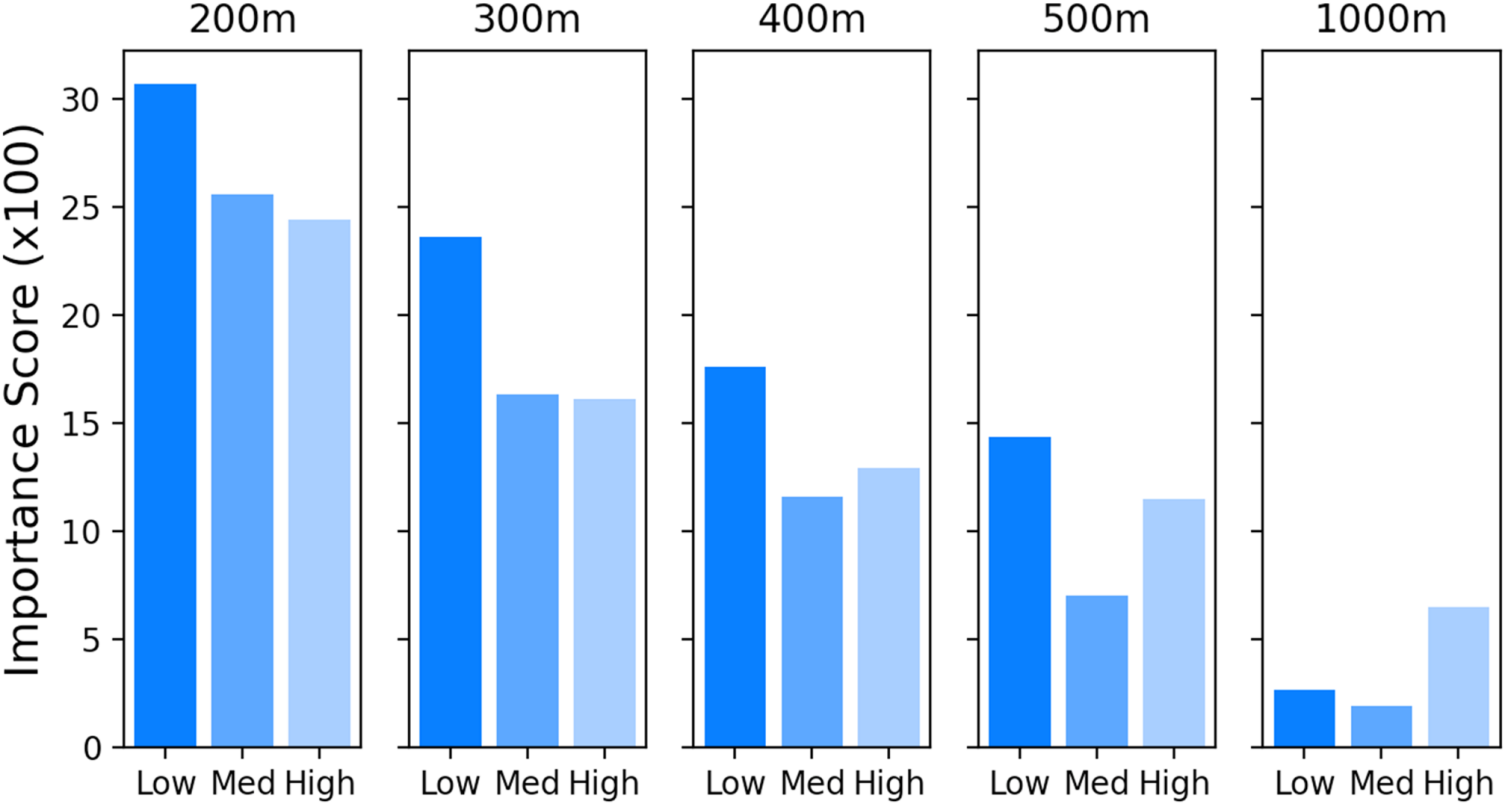
**Importance of panel density moderated by income**.The importance of solar panel density around an address is computed across different income groups, with density calculated at radii of 200 m, 300 m, 400 m, 500 m, and 1 km. The point value contribution to model performance of the density variable multiplied by 100 is shown on the y-axis, and is compared across income subgroups and five density calculation radii. The importance of panel density within small radii is most pronounced in low-income groups. For larger radii, the relevance is reduced and the income relationship reversed (see Supplementary Figure S17).

Figures 2 and 3 show that proximity to other solar panels is the most important predictor for having a solar panel installed and that this importance decreases the further away the other panels are. In order to test for potentially heterogeneous effects, i.e. whether this effect is more pronounced in some social groups than in others, we split that data in accordance with several socio-economic categories. Binning our data into several income brackets reveals a nonlinear interaction of household income and distance over which panel density is calculated (again using the differenced density calculation here) (Fig. 4). Specifically, we define three income brackets based on the distribution of this feature (see Supplementary Figure S15). We define the lowest income bracket to be less than $42,000/year, mid-income between $42,000 and $80,000/year, and high income as more than $80,000/year. Figure 4 shows the permuted importance scores across binned household incomes for five radii (200 m, 300 m, 400 m, 500 m, and 1000 m), again calculated using contribution to the area under the precision-recall curve. Across all income groups, the importance of panel density decreases with larger radii. However, the importance of high panel density at small radii (200 m to 500 m) is most pronounced in the low-income group. For larger radii (1000 m), this relation becomes insignificant. The extraction of a clear relation is further complicated by the fact that in this case high-income groups might be more likely to live in less densely populated areas. These results suggest that the relationship between distance and likelihood of having a panel is moderated by income. Splitting by median home value shows comparable effects across different groups (see Supplementary Figure S16).

## Discussion

Our results confirm findings from the literature about the importance of proximity to other solar panels in solar panel adoption. Using high-resolution data at the household level for the city of Fresno, we further find evidence that it is those buildings in closest proximity that best predict the likelihood of solar panels being installed at an address. This conclusion supports a qualitative study showing the significant effect of neighbours on installation^7^, as well as the importance of close proximity^2,3,6^ and nonlinear decrease in peer effects as distance increases over 200 m^5,19^. We are able to quantify this effect, finding that the neighbour effect is more influential at 200 m and that this effect decreases non-linearly as distance from an installed panel increases. Our data show an exponential decay in the importance of proximity, indicating that those neighbours that are closest are the most influential in determining solar panel adoption behaviour. At larger distances there is still an important influence of the existence of solar panels on the decision to install a panel, but this influence decays with an exponential decay rate of 210 m.

In contrast to those results found by Müller and Rode for Germany^19^, we do not find that high socio-economic status is a consistently strong predictor of being an adopter in Fresno, USA. In contrast, we find that proximity exerts the strongest influence in the low-income bracket of our dataset. Similar to others in the literature, our study is here limited by the granularity of our socio-economic and demographic data which are only available at the level of the census-tract. Furthermore, we lack data on other forms of potential social networks outside of residential proximity and school districts. To the extent that individualized data are available, further study of the effect of other types of social networks, such as interest groups, sports organizations, or cultural or religious communities, would have the potential to better distinguish between the effect of social networks and that of proximity.

Methodologically, we are limited by the problem of class imbalance in our data. As discussed in the “Data and methods” section, only about 3% of addresses have a solar panel assigned. This affects the performance of our classifier, as it has very little information on those addresses that do have panels that it can use to predict the existence of a panel on an unseen address. We implement several statistical mechanisms to address this issue (see “Data and methods” section). Even with this limitation, our results demonstrate the utility of machine learning methodologies in descriptive analyses, especially in cases where nonlinear relationships are likely to exist.

Our results suggest that most effectively expanding the use of solar panels could rely on targeting uptake in areas with few or no solar panels, rather than encouraging spread where panels already exist. Cumulative usage might be improved by ‘seeding’ panels where they are scarce, or in densely inhabited areas, to take advantage of the influence of close proximity that we find.

## Data and methods

We construct a novel dataset that provides information on both socio-economic characteristics and solar panel installation information for each address in the city of Fresno, USA, from several sources of geo-located data.

### Solar panel data

The first is a dataset of 601 satellite images with corresponding geo-located border vertices for more than 19,000 solar panels across four cities in California in 2014^12^. The centroid of each border area is used for the panel’s geolocation. We select those images and panels located in Fresno, California, and the surrounding area, which is 412 images and 14,803 solar panels. The other cities for which geo-located panel data are available are not included due to a lack of localized address data. The satellite images are used here to create a bounding box for the area to be included in the analysis (Fig. 1). Just 2% of panels are explicitly marked as residential, and the rest are not classified. As they are derived from satellite images, it is likely that both commercial and industrial buildings are included, as the satellite images cover all buildings in an area indiscriminately.

### Geo-located data for the city of Fresno

Data from the County of Fresno and Institute for Education Science provide information on the geographic boundaries of Fresno’s 13 county school districts, 158 census tracts, and information on the geolocation of the 292,060 of Fresno’s addresses located in the area covered by the satellite images^13,14^.

### Socio-economic and demographic data

Household demographic, social, and economic data for all 158 census tracts of Fresno are provided by the American Community Survey (ACS) Demographic and Housing 5 Year Estimates from 2009–2013^15^. These data are aggregated at the census tract level and provide information on median household income, median house value, median monthly rent, unemployment, tract area, percentage of the population identifying as a single race, the number of households receiving SNAP benefits, education, total population, total households, veteran status, school district, and percentage of homes occupied (described in Supplementary Table S11). In preliminary analyses, we included all variables available in the ACS, but excluded variables that were insignificant to the analysis and unimportant to the prediction of the outcome in final tables and figures.

### Mapping socio-economic and demographic data to addresses

Address data are linked to census-tract level data by geo-locating each address into the census tract in which it exists. Similarly, school district data are matched with address data by looking up in which school district the addresses lie.

### Mapping panels to addresses

We overlay the geolocations of 14,803 solar panels in the city of Fresno, California, derived from a dataset of satellite images with geo-locations of all of Fresno’s addresses, as provided by the County of Fresno. To identify those addresses that have a solar panel, we measure the distances between all solar panels and addresses, and between all addresses and all other addresses. A solar panel is assigned to a house if it is both the closest solar panel to that address, and not unreasonably far (defined as more than 1 km) away. This ensures that panels can also be associated with an address if they are not installed directly on the building, without erroneously assigning panels to addresses to which they do not correspond (the case of solar farms, for instance). As many of the satellite images show the possibility of addresses having more than one solar panel, addresses can be assigned multiple solar panels, if these panels are closer to each other than they are to the next closest address. After geo-restricting to the city boundaries of Fresno and that area covered by the satellite images, we obtain a dataset containing 9158 addresses to which panels are assigned out of 292,060 addresses in total (see Fig. 1).

### Determining solar panel density

To build our feature of interest, the density of solar panels around an address, we first count the number of solar panels within variable radii of an address, excluding any panels at that address itself. These radii range from 200 m to 1200 m, increasing by 100 m increments. This panel density is then normalized by address density, which we similarly derive by counting the other addresses (both with and without panels) within the same radius of that address. For Figs. 3 and 4, at each radius, we subtract this normalized panel density of the previous radius from that of the current radius, in order to isolate the gains in importance from just the 100 m increase over the previous radius and its associated panel density.

### Building a classifier

We build a tree-based classifier that predicts if a particular address has a solar panel based on those features included in our dataset. To this end, two classes are defined: addresses at which there is a solar panel, and addresses without. We test three different commonly used classifiers: Random Forest, XGBoost, and AdaBoost. Each of these algorithms distinguishes between these two classes based on the socioeconomic, demographic, and school district information provided in our dataset. We fit each of the three algorithms ten times, each time replacing the density feature by the next radius over which density is calculated. For each of the ten, the Random Forest Classifier is fit with bootstrapping and ‘balanced’ class weight, in order mitigate the class imbalance (detailed below). XGBoost is fit with the scale positive weight set to the sum of negative instances (no panel addresses) divided by the sum of positive instances (addresses having panels), again to manage the class imbalance problem. Finally, AdaBoost is fit with a Decision Tree Classifier as its base estimator and SAMME.R boosting algorithm (for speed of convergence), which we find to exhibit the best performance. After fitting the classifiers, we analyse those features that are most influential in accurately predicting if an address has a solar panel.

### Addressing data imbalance problem

Only 3% of addresses in the dataset have a corresponding panel, implying a large class imbalance problem. Given the comparatively little information available on the positive class (addresses with a panel) it is difficult for a classifier to accurately predict this class, which is the class of interest. Therefore, before applying any of the three tree-based models tested, we test Random Over and Under Sampling, as well as the Synthetic Minority Oversampling Technique (SMOTE)^20^ to augment the minority class or diminish the majority class, in order to improve the performance of the classifier. The largest improvement in performance is seen for the Synthetic Minority Oversampling Technique. This technique artificially generates new samples in the minority class by first randomly selecting a sample from that class. The synthetic example is created by randomly selecting a sample in the feature space between the nearest neighbours of that sample for each feature. This technique is applied to the dataset before the implementation of all algorithms.

### Selecting evaluation metrics

The class imbalance problem also requires the adjustment of evaluation metrics. Given the 3% representation of the minority class, optimizing the algorithm for overall accuracy (number of correct predictions out of total predictions) could easily result in a classifier that achieves 97% overall accuracy by only predicting addresses to be of the majority class. We therefore calculate two other evaluation metrics along with the most commonly used evaluation procedures (accuracy and the confusion matrix): the area under the receiver operating characteristic curve (AUC ROC) and the area under the precision-recall (AU P-R Curve). Both of these metrics are better suited for cases of class imbalance problems as they consider the True Positive Rate (how many positive cases are correctly predicted out of total positive cases). Unlike the confusion matrix, which presents the number of correctly and incorrectly predicted addresses with panels and addresses without panels for a decision threshold of 0.5, both the AUC-ROC and AU P-R metrics provide an aggregate measure of performance which is invariant to the decision threshold, as they are calculated for all possible thresholds.

While the combination of these two metrics allows us to more holistically evaluate our trained algorithm (henceforth referred to as the model), the area under the precision-recall curve is used as the primary evaluation metric as it is most suitable for imbalanced class problems. In contrast to the AUC-ROC where the secondary axis is the False Positive Rate (number of incorrectly identified negative cases out of all negatives), the AU P-R Curve considers precision (the number of true positives of all predicted positives). This puts preferential importance on the prediction of the positive class, in our case, leading us to prioritize the correct prediction of addresses that have panels. Further, Davis & Goadrich show that an algorithm will dominate in AUC-ROC space if and only if it dominates in AU P-R Curve scoring as well^20^.

After testing, the best performing algorithm by these metrics was the AdaBoost Classifier with a decision tree classifier as its base estimator (Supplementary Discussion 1). We therefore base our results on this model. All evaluation metrics are computed comparing the predicted outcomes and the true outcomes in the test set, which comprises a randomly selected 30% of the data, withheld from the training phase.

### Evaluating feature importance

We evaluate the features that are most important in determining if an address has a panel using feature importance. Unlike in parameterized methodologies, the *pattern* of the relationship between the features and outcome is not defined (and can therefore take any form). The by-product of not specifying this functional form is a trade-off between interpretability and performance. It is therefore only possible to specify the *strength* of dependency between each feature and the outcome, in the form of feature importance, as well as the direction of this correlation^16^. Feature importances calculated using the weighted mean of individual trees’ performance at each variable node (or information gain-based measures, such as Gini importance or entropy) have been shown to be biased in their estimation of true importance of a feature to the model, inflating the influence of high-cardinality categorical or large-scale continuous variables^21^. We therefore choose to employ “permutation accuracy importance” as our measure of feature importance. In this methodology, feature importance is determined by randomly shuffling the rows of each feature column so as to break their association with the outcome, one at a time. This permuted variable is then combined with the other unpermuted features, and the model is re-estimated. The feature importance is reported as the average loss in the selected performance metric due to the permutation of that feature over all iterations. This method corrects for the feature importance bias towards categorical and high cardinality variables^22^.

The feature importances presented are the average contribution of that variable to the model’s performance, defined using the area under the precision-recall curve as our performance metric. To obtain this value, the permuted importance for each feature is calculated, which gives a point value estimate for the mean contribution to performance for each feature. This calculation is in line with the assumptions outlined in Fisher et al. for the calculation of unbiased feature importances to be compared across models^23^.

### Direction of influence

The hypothesized influence of proximity to a solar panel is confirmed by examining the direction of correlation between each variable and the outcome. Because the feature importance scores presented describe the improvement in the performance of the algorithm due to the inclusion of that variable, but not the *direction* of influence of that variable on the outcome, we calculate the pair-wise Pearson’s correlation between all variables and the outcome (Supplementary Table S12).

### Assessing feature importance significance

The significance of the permuted feature importance scores are examined by calculating p-values. To compute these, we first permute each feature over 50 iterations as suggested by Altmann et al.^24^. We then compute the two-tailed t-statistic and p-value from the estimated accuracy score and standard deviation provided, in order to determine at what level of significance each feature’s mean feature importance is determined to be different from zero. No difference from zero, or a computed difference insignificant at the 0.1, 0.05, or 0.01 level of significance would indicate that including the feature in the model does not improve the model’s predictions as that feature does not carry any predictive power. P-values are provided for the main model in Supplementary Table S10.

## Supplementary Information

Supplementary Information.

## Data Availability

The dataset was constructed from publicly available data (ref. [12–15]). The combined dataset with information on both socio-economic characteristics and solar panel installation for each address in the city of Fresno, USA, is publicly available at 10.5281/zenodo.4676430 (ref. [25]).

## Code availability

The code used for the analysis and plots is available at 10.5281/zenodo.4676430.
