# Supplementary material for: Decay radius of climate decision for solar panels in the city of Fresno, USA
Source: Sci Rep. 2021 Apr 21;11:8571. doi: 10.1038/s41598-021-87714-w (PMC8060319; doi:10.1038/s41598-021-87714-w)
Supplement: Supplementary file 1 — Supplementary Information. [file 41598_2021_87714_MOESM1_ESM.pdf]

Supplementary Information for:

Decay radius of climate decision for solar panels in the city of Fresno, USA

Kelsey Barton-Henry[a], Leonie Wenz[a, c, d, *], Anders Levermann[a, b, e].

(a) Potsdam Institute for Climate Impact Research, Potsdam, Germany;

(b) Institute of Physics, Potsdam University, Potsdam, Germany;

(c) Mercator Research Institute on Global Commons and Climate Change, Berlin, Germany;

(d) Department of Agriculture and Resource Economics, University of California, Berkeley, USA;

(e) Columbia University, New York, NY, USA.

* corresponding author: leonie.wenz@pik-potsdam.de.

Overview: This document provides supplementary information for Barton-Henry, Wenz, and Levermann (2021). This document is structured in the following sections:

1. A short description of all features included in the model.
2. A discussion of model performance for all tested models (AdaBoost, Random Forest, and XGBoost). A subsection for each model is provided, containing the following:
    a. A figure displaying feature importance scores for the normalized density variables at all radii.
    b. A table of performance metrics (Total Accuracy, the Area Under the Receiver Operating Characteristic Curve, and the Area Under the Precision-Recall Curve) for models built at all density radii.
    c. Three confusion matrices for models built with panel density variables corresponding to 200m, 500m, and 1000m radii.
3. Feature importance scores by variable, for the main AdaBoost model re-calculated using all density variable radii.
4. An evaluation of all tested models without the inclusion of panel density variables.
5. A robustness check in which OLS models are built with normalized panel density radii of 200m, 500m, and 1000m.
6. A comparison of feature importances when panel density is averaged over census tract.
7. Presentation of feature importances without the subtraction of the previous radius.
8. An analysis of the decay in panel density importance over increasing radii when the data is subset by the number of households in the census tract and tract area.
9. Presentation of calculated p-values for variables calculated in Figure 3.
10. The distribution of household income in the dataset, by which the data is subset.

11. An analysis of normalized panel density importance when data is subgrouped by median home value.
12. An analysis of normalized panel density importance when data is subgrouped by income.
13. Descriptive statistics for census tract level features.
14. Presentation of the correlation score and direction for all normalized panel density variables with the outcome.

Section 1. Overview of all features included in the model.

Name	Description
Panel density	Normalized density of panels within selected radius of address
Income	Median household income over census tract
Med. H. Val	Median home value over census tract
Med. Rent	Median monthly rent for renter-occupied-units in tract
Unemployed	Percent unemployment in tract
Tract area	Area of census tract
Single race	Percent of total tract population identifying as a single race
Tract	Identifier of census tract
SNAP	Number of households having received SNAP benefits in last 12 months
Education	Percent of individuals 25+ with bachelor's or higher in tract
Total pop	Total population of census tract
Total HH	Total households in census tract
Veteran	Percent of civilian 18+ population with veteran status in tract
School dist	School district in which the address lies
Occupied	Percent of total housing units occupied in tract

Table S1. *Names and short descriptions of all features included in the model. These features are consistent across all models, with the exception of Panel Density, which varies with the radius over which panel density is calculated.*

Section 2. Performance evaluations for all tested models.

Supplementary Discussion 1

AdaBoost

The AdaBoost model was chosen as it was evaluated to be the most robust model on the relevant evaluation metrics (Area Under the Receiver Operating Characteristic and Area Under the Precision-Recall Curve) as well via the Confusion Matrix. Bar plots of the normalized density feature importances are presented with model performance metrics across the 10 models below for the three models tested: AdaBoost, Random Forest, and XGBoost. Confusion matrices are provided for the models with density calculated at 200m, 500m and 1000m radii. All models are estimated using the normalized panel density calculated each radius, with the panel density of the previous radius subtracted.

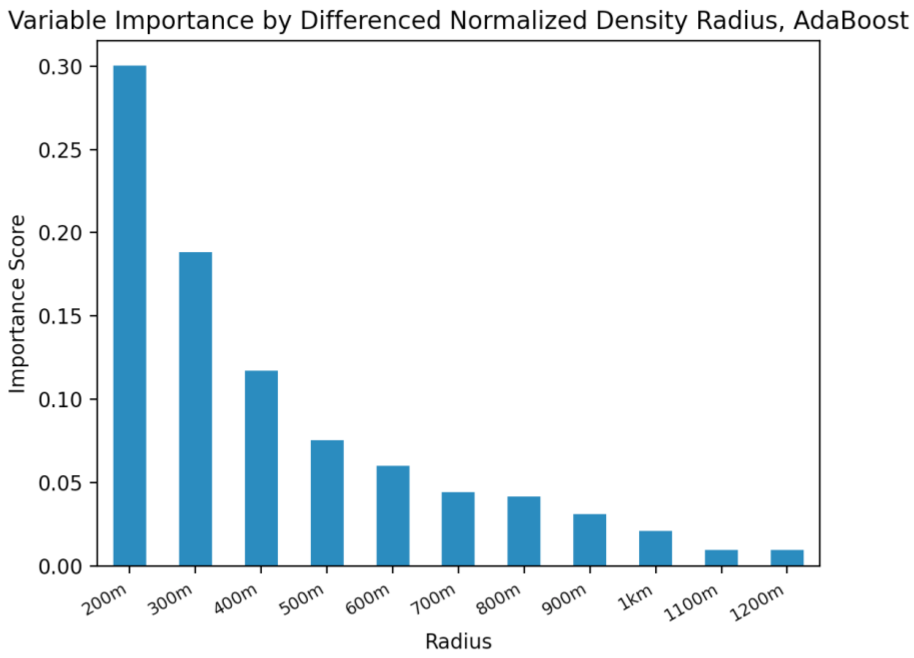

Figure S1. *Feature importance scores for normalized density variables at all radii, comparing across all models using the AdaBoost algorithm.*

Model Performance Metrics: AdaBoost

Density Model	Total Acc.	AUC ROC	AU P-R Curve
200m	0.732338	0.788597	0.473229
300m	0.745999	0.736201	0.410160
400m	0.728891	0.723194	0.403007
500m	0.710219	0.722939	0.410805
600m	0.700564	0.717431	0.409059
700m	0.708689	0.709981	0.397322
800m	0.709318	0.705785	0.392457
900m	0.682143	0.705485	0.404361
1km	0.694058	0.704511	0.397896
1100m	0.679255	0.696171	0.395568
1200m	0.672841	0.696510	0.398888

Table S2. *Performance metrics (Total Accuracy, the Area Under the Receiver Operating Characteristic Curve, and the Area Under the Precision-Recall Curve) and the corresponding radii for each model employing the AdaBoost algorithm.*

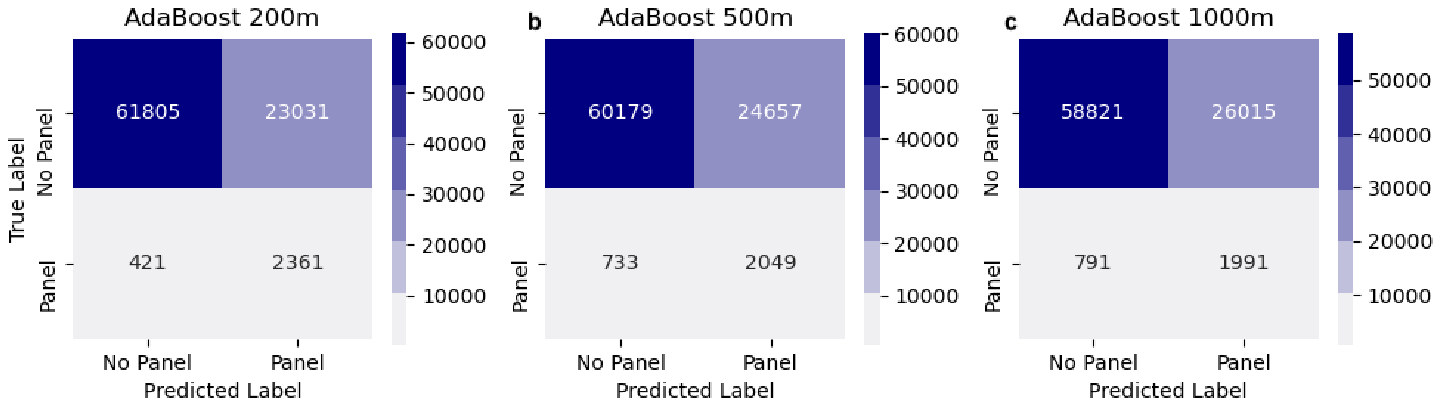

Figure S2. *Confusion matrices for the AdaBoost models run with the 200m, 500m, and 1000m density radii, showing the number of correctly and incorrectly classified addresses of each type (Panel and No Panel). All confusion matrices are computed for a decision threshold of 0.5.*

Random Forest

While it demonstrated the best overall performance metrics, the Random Forest model was discarded because while it did well in identifying addresses without panels, its performance on the minority class sacrificed its performance on the majority class: comparatively to the AdaBoost model, it misclassified a larger percentage of those houses with panels.

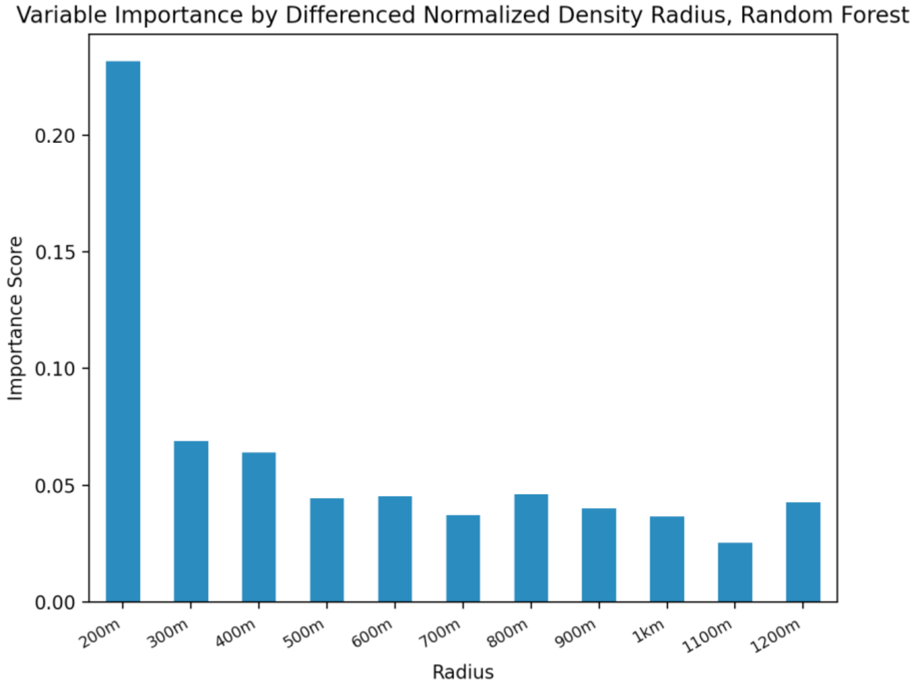

Figure S3. *Feature importance scores for normalized density variables at all radii, comparing across all models using the Random Forest algorithm.*

Model Performance Metrics: Random Forest			
**Density Model**	**Total Acc.**	**AUC ROC**	**AU P-R Curve**
200m	0.813817	0.716464	0.362704
300m	0.738227	0.644401	0.311997
400m	0.729256	0.640290	0.311503
500m	0.721096	0.629992	0.303872
600m	0.710755	0.627781	0.306248
700m	0.711212	0.613415	0.290214
800m	0.709443	0.615631	0.293490
900m	0.706989	0.613147	0.291917
1km	0.704536	0.616747	0.297039
1100m	0.7051512	0.603854	0.282564
1200m	0.702629	0.616110	0.297242

Table S3. *Performance metrics (Total Accuracy, the Area Under the Receiver Operating Characteristic Curve, and the Area Under the Precision-Recall Curve) and the corresponding radii for each model employing the Random Forest algorithm.*

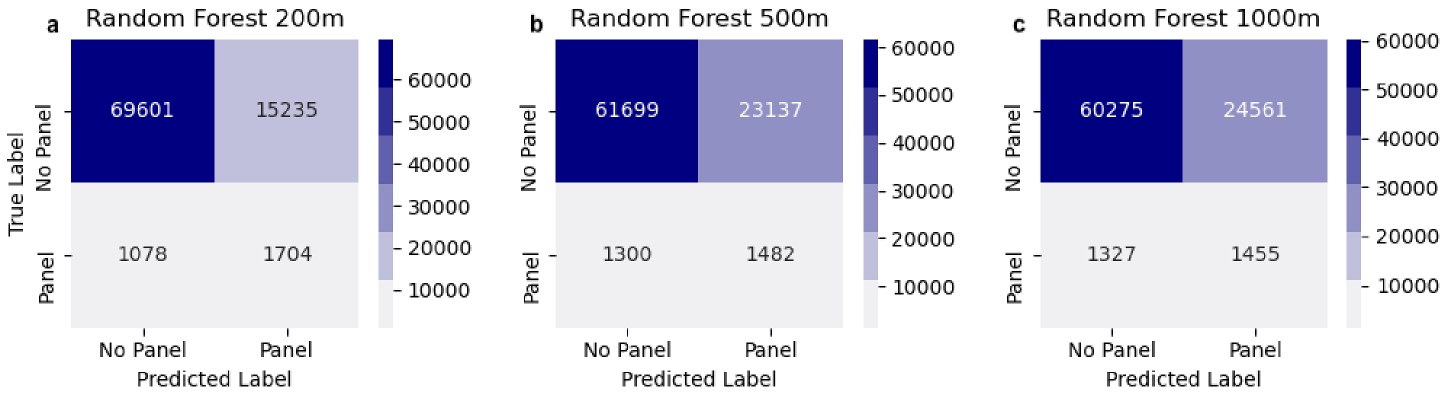

Figure S4. *Confusion matrices for the Random Forest models run with the 200m, 500m, and 1000m density radii, showing the number of correctly and incorrectly classified addresses of each type (Panel and No Panel). All confusion matrices are computed for a decision threshold of 0.5.*

XGBoost

The XGBoost algorithm was discarded for demonstrating the poorest performance metrics of the three algorithms tested, as shown in the following figures and tables.

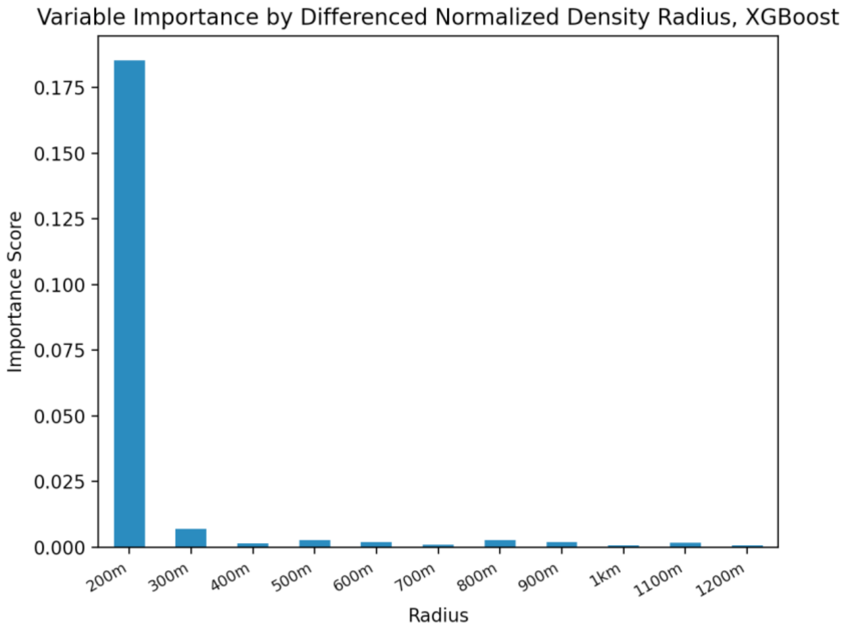

Figure S5. *Feature importance scores for normalized density variables at all radii, comparing across all models using the XGBoost algorithm.*

Model Performance Metrics: XGBoost

Density Model	Total Acc.	AUC ROC	AU P-R Curve
200m	0.386964	0.680475	0.521528
300m	0.135132	0.551647	0.515907
400m	0.095768	0.530972	0.514805
500m	0.092926	0.529331	0.514574
600m	0.082859	0.524654	0.514934
700m	0.080623	0.522977	0.514356
800m	0.082517	0.524129	0.514569
900m	0.083647	0.524365	0.514230
1km	0.070145	0.517567	0.514175
1100m	0.072873	0.519149	0.514402
1200m	0.071835	0.518613	0.514384

Table S4. *Performance metrics (Total Accuracy, the Area Under the Receiver Operating Characteristic Curve, and the Area Under the Precision-Recall Curve) and the corresponding radii for each model employing the Random Forest algorithm.*

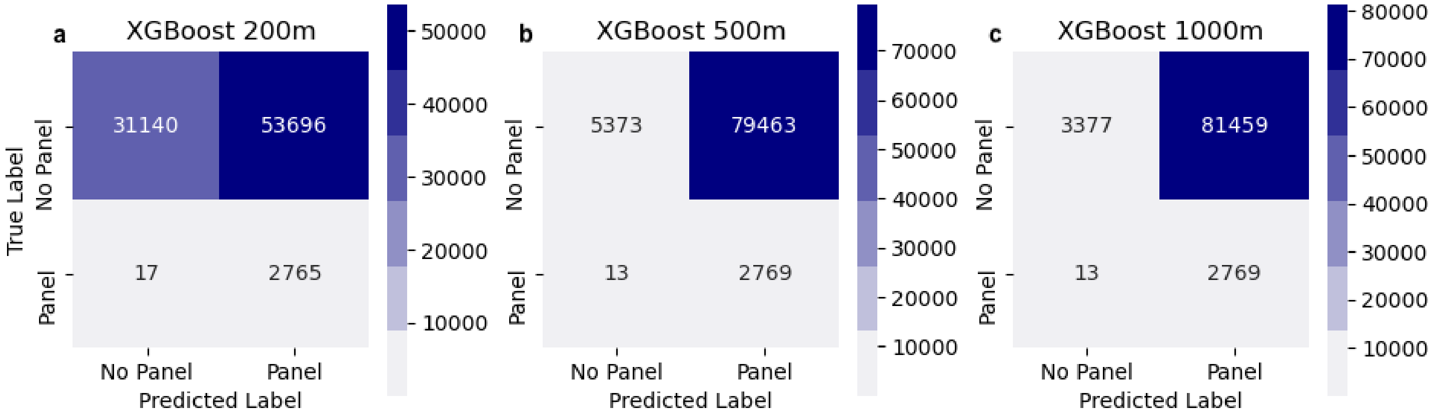

Figure S6. *Confusion matrices for the XGBoost models run with the 200m, 500m, and 1000m density radii, showing the number of correctly and incorrectly classified addresses of each type (Panel and No Panel). All confusion matrices are computed for a decision threshold of 0.5.*

Section 3. Feature importance scores by variable, for the main AdaBoost model re-calculated using all density variable radii.

Feature	200m	300m	400m	500m	600m	700m
Panel density	30.7426	18.8312	14.1525	7.7391	5.1845	4.1772
Income	0.0664	0.1849	0.1780	0.2602	0.0453	0.1190
Med. H. Val	0.1172	-0.1616	0.2221	0.1470	0.3289	0.3599
Med. Rent	0.0113	-0.0789	0.0346	-0.0325	-0.0834	0.0398
Unemployed	-0.0072	-0.0570	0.0038	-0.0454	-0.0214	-0.0047
Tract area	0.2711	0.1654	0.2470	0.1977	0.2178	0.3110
Single race	0.1286	0.0959	0.1275	0.0722	0.0661	0.0908
Tract	-0.0058	0.0000	0.0000	0.0000	0.0000	0.0000
SNAP	0.0000	-0.0070	0.0347	0.0000	0.2094	0.0396
Education	0.0000	-0.0599	0.1173	0.0000	0.0000	0.0000
Total pop	-0.0057	0.0000	0.0000	0.0000	0.0000	0.0000
Total HH	0.0000	0.1162	0.0922	-0.0733	-0.0239	0.0847
Veteran	0.0000	0.0000	-0.0117	0.0585	0.0521	0.0728
School dist	0.0180	0.0190	0.0144	0.0190	0.0112	0.0153
Occupied	0.0000	0.0000	0.0000	0.0000	-0.0088	0.0783

Feature	800m	900m	1000m	1100m	1200m
Panel density	4.6162	3.4269	1.6573	1.6393	0.8634
Income	0.3543	0.0509	-0.1881	-0.2809	0.4344
Med. H. Val	0.4603	1.196	1.2513	1.1363	1.2964
Med. Rent	-0.0306	-0.0130	-0.1066	-0.1109	-0.1439
Tract area	0.1897	0.2243	0.4269	0.1978	0.2106
Single race	-0.1737	0.2085	0.1924	0.2471	-0.2519
Tract	0.0000	0.0006	-0.0237	0.1266	-0.4713
SNAP	0.03918	0.3000	0.1429	0.0340	0.730
Education	0.2129	-0.0038	0.0000	0.0000	-0.3965
Total pop	0.0000	0.0416	-0.0140	-0.0721	-0.1516
Total HH	-0.1189	0.0000	0.0000	0.2975	0.1521
Veteran	0.0592	-0.0126	0.0773	0.2975	0.2081
School dist	5.0234	4.6713	0.0002	0.0001	-0.0001
Occupied	-0.0332	-0.0003	0.1116	0.167	-0.0790

Table S5. *Permuted feature importance scores by variable shown as the point value contributions to performance by each feature in the model averaged over 50 permutations, multiplied by 100. Importance scores from all models, each with panel density calculated over radii from 200m to 1200m increasing at 100m increments, are shown. For each model, panel density consistently contributes the largest gains in performance.*

Section 4. An assessment of all tested models without the inclusion of panel density variables.

Supplementary Discussion 2

We provide the confusion matrices along with overall performance metrics (Total Accuracy, the Area Under the Receiver Operating Characteristic Curve, and the Area Under the Precision-Recall Curve) for all models, but built without the inclusion of density variables. Across all models, we find that model performance suffers significantly, especially when compared with the models with the smallest radius density calculations, further indicating the importance of the density variables to the accurate prediction of the existence of panels at a particular address.

Model Performance Metrics: No Density Variables

Model	Total Acc.	AUC ROC	AU P-R Curve
AdaBoost	0.626789	0.686287	0.409477
Random Forest	0.674005	0.693113	0.394666
XGBoost	0.102468	0.530433	0.510793

Table S6. *Performance metrics for all models when built without the inclusion of panel density variables (Total Accuracy, the Area Under the Receiver Operating Characteristic Curve, and the Area Under the Precision-Recall Curve).*

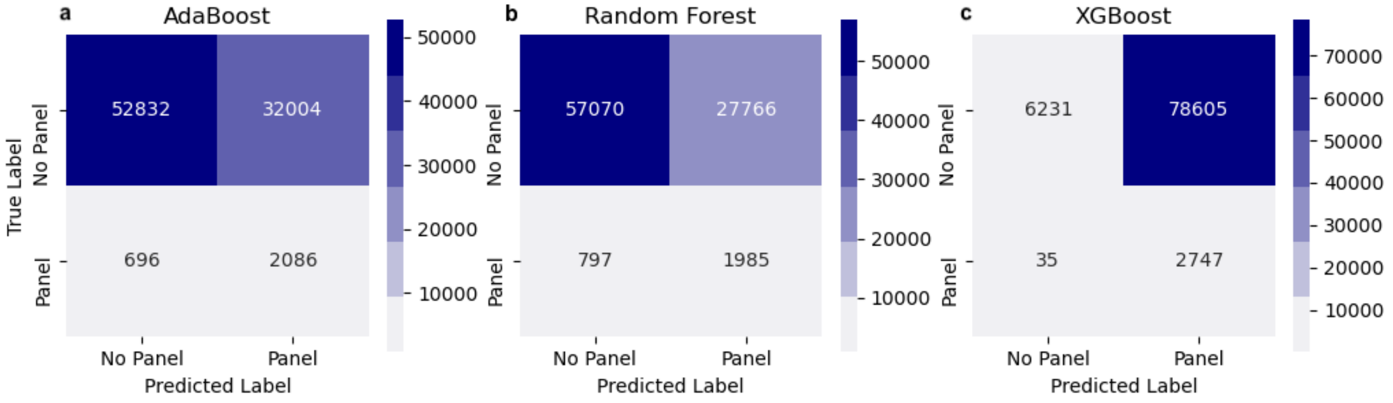

Figure S7. *Confusion matrices for all models run without density variables, showing the number of correctly and incorrectly classified addresses of each type (Panel and No Panel). All confusion matrices are computed for a decision threshold of 0.5.*

Section 5. A robustness check in which OLS models are built with normalized panel density radii of 200m, 500m, and 1000m.

200m OLS Results

**Dep. Variable:**	has_panel	**R-squared:**	0.074
**Model:**	OLS	**Adj. R-squared:**	0.074
**Method:**	Least Squares	**F-statistic:**	865.9
**Date:**	Mon, 17 Aug 2020	**Prob (F-statistic):**	0.00
**Time:**	11:38:24	**Log-Likelihood:**	1.0709e+05
**No. Observations:**	292060	**AIC:**	-2.141e+05
**Df Residuals:**	292032	**BIC:**	-2.138e+05
**Df Model:**	27		

	**coef**	**std err**	**t**	**P>|t|**	**[0.025**	**0.975]**
**Panel Density 200m**	4.4368	0.034	129.946	0.000	4.370	4.504
**Total pop**	0.0353	0.007	4.898	0.000	0.021	0.049
**Unemployed**	0.0118	0.003	4.038	0.000	0.006	0.018
**Income**	0.0046	0.004	1.126	0.260	-0.003	0.012
**SNAP**	-0.0040	0.003	-1.617	0.106	-0.009	0.001
**Med. H. Val**	0.0213	0.004	5.000	0.000	0.013	0.030
**Med. Rent**	0.0073	0.003	2.412	0.016	0.001	0.013
**Total HH**	-0.0372	0.055	-0.672	0.502	-0.146	0.071
**Education**	0.0128	0.003	3.882	0.000	0.006	0.019
**Veteran**	0.0010	0.002	0.397	0.691	-0.004	0.006
**Single race**	0.0057	0.005	1.145	0.252	-0.004	0.015
**Occupied**	-0.0058	0.003	-1.664	0.096	-0.013	0.001
**Tract**	-0.0041	0.002	-1.907	0.056	-0.008	0.000
**Tract area**	-0.0666	0.013	-5.129	0.000	-0.092	-0.041
**American Union Elem**	-0.0494	0.013	-3.854	0.000	-0.075	-0.024
**Clovis Unified**	-0.0122	0.005	-2.520	0.012	-0.022	-0.003
**Central Unified**	-0.0142	0.005	-2.963	0.003	-0.024	-0.005
**Fresno Unified**	-0.0122	0.005	-2.676	0.007	-0.021	-0.003
**Fowler Unified**	-0.0415	0.007	-6.097	0.000	-0.055	-0.028
**Pacific Union Elem**	0.0992	0.069	1.446	0.148	-0.035	0.234
**Orange Center Elem**	-0.0192	0.008	-2.402	0.016	-0.035	-0.004
**Sanger Unified**	-0.0130	0.005	-2.476	0.013	-0.023	-0.003
**Washington Colony Elem**	-0.0270	0.024	-1.136	0.256	-0.073	0.020
**West Fresno Elem**	-0.0061	0.005	-1.129	0.259	-0.017	0.005
**West Park Elem**	-0.0344	0.009	-3.998	0.000	-0.051	-0.018

**Omnibus:**	274094.128	**Durbin-Watson:**	1.809
**Prob(Omnibus):**	0.000	**Jarque-Bera (JB):**	10674539.105
**Skew:**	4.654	**Prob(JB):**	0.00
**Kurtosis:**	31.116	**Cond. No.**	452.

Table S7. *A naïve OLS model is built using panel density calculated at a radius of 200m. The panel density variable has the largest coefficient and is highly significant.*

500m OLS Results

**Dep. Variable:**	has_panel	**R-squared:**	0.052
**Model:**	OLS	**Adj. R-squared:**	0.052
**Method:**	Least Squares	**F-statistic:**	593.9
**Date:**	Mon, 17 Aug 2020	**Prob (F-statistic):**	0.00
**Time:**	11:38:26	**Log-Likelihood:**	1.0365e+05
**No. Observations:**	292060	**AIC:**	-2.072e+05
**Df Residuals:**	292032	**BIC:**	-2.069e+05
**Df Model:**	27		

	**coef**	**std err**	**t**	**P>|t|**	**[0.025**	**0.975]**
**Panel Density 500m**	3.6352	0.037	98.443	0.000	3.563	3.708
**Total Pop**	0.0088	0.007	1.196	0.232	-0.006	0.023
**Unemployed**	0.0075	0.003	2.541	0.011	0.002	0.013
**Income**	-0.0016	0.004	-0.396	0.692	-0.010	0.006
**SNAP**	-0.0025	0.003	-0.979	0.328	-0.007	0.002
**Med. H. Val**	0.0074	0.004	1.690	0.091	-0.001	0.016
**Med. Rent**	0.0001	0.003	0.035	0.972	-0.006	0.006
**Total HH**	0.1142	0.056	2.037	0.042	0.004	0.224
**Education**	0.0066	0.003	1.969	0.049	3.06e-05	0.013
**Veteran**	-0.0012	0.003	-0.496	0.620	-0.006	0.004
**Single Race**	-0.0076	0.005	-1.511	0.131	-0.017	0.002
**Occupied**	-0.0002	0.004	-0.056	0.956	-0.007	0.007
**Tract**	-0.0016	0.002	-0.738	0.460	-0.006	0.003
**Tract area**	0.0020	0.013	0.153	0.879	-0.024	0.028
**American Union Elem**	-0.0028	0.013	-0.213	0.831	-0.028	0.023
**Clovis Unified**	-0.0023	0.005	-0.476	0.634	-0.012	0.007
**Central Unified**	-0.0011	0.005	-0.219	0.826	-0.011	0.008
**Fresno Unified**	-0.0003	0.005	-0.072	0.942	-0.009	0.009
**Fowler Unified**	-0.0478	0.007	-6.944	0.000	-0.061	-0.034
**Pacific Union Elem**	0.1308	0.069	1.883	0.060	-0.005	0.267
**Orange Center Elem**	-0.0003	0.008	-0.035	0.972	-0.016	0.016
**Sanger Unified**	0.0016	0.005	0.309	0.757	-0.009	0.012
**Washington Colony Elem**	0.0122	0.024	0.510	0.610	-0.035	0.059
**West Fresno Elem**	0.0020	0.005	0.364	0.716	-0.009	0.013
**West Park Elem**	-0.0146	0.009	-1.673	0.094	-0.032	0.003

**Omnibus:**	280412.863	**Durbin-Watson:**	1.775
**Prob(Omnibus):**	0.000	**Jarque-Bera (JB):**	8971685.019
**Skew:**	4.938	**Prob(JB):**	0.00
**Kurtosis:**	28.293	**Cond. No.**	452.

Table S8. *A naïve OLS model is built using panel density calculated at a radius of 500m. The panel density variable has the largest coefficient and is highly significant.*

1000m OLS Results

**Dep. Variable:**	has_panel	**R-squared:**	0.038
**Model:**	OLS	**Adj. R-squared:**	0.038
**Method:**	Least Squares	**F-statistic:**	428.1
**Date:**	Mon, 17 Aug 2020	**Prob (F-statistic):**	0.00
**Time:**	11:38:29	**Log-Likelihood:**	1.0151e+05
**No. Observations:**	292060	**AIC:**	-2.030e+05
**Df Residuals:**	292032	**BIC:**	-2.027e+05
**Df Model:**	27		

	**coef**	**std err**	**t**	**P>|t|**	**[0.025**	**0.975]**
**Panel Density 1000m**	0.7484	0.010	72.859	0.000	0.728	0.769
**Total pop**	0.0121	0.007	1.636	0.102	-0.002	0.027
**Unemployed**	0.0009	0.003	0.309	0.757	-0.005	0.007
**Income**	-0.0028	0.004	-0.674	0.501	-0.011	0.005
**SNAP**	-0.0036	0.003	-1.397	0.162	-0.009	0.001
**Med. HH. Val**	0.0090	0.004	2.025	0.043	0.000	0.018
**Med. Rent**	-0.0036	0.003	-1.170	0.242	-0.010	0.002
**Total HH**	0.2956	0.057	5.214	0.000	0.184	0.407
**Education**	-3.356e-05	0.003	-0.010	0.992	-0.007	0.007
**Veteran**	0.0007	0.003	0.263	0.792	-0.004	0.006
**Single Race**	-0.0259	0.005	-5.109	0.000	-0.036	-0.016
**Occupied**	-0.0059	0.004	-1.645	0.100	-0.013	0.001
**Tract**	-0.0029	0.002	-1.329	0.184	-0.007	0.001
**Tract area**	-0.0003	0.013	-0.025	0.980	-0.026	0.026
**American Union Elem**	0.0234	0.013	1.786	0.074	-0.002	0.049
**Clovis Unified**	0.0139	0.005	2.788	0.005	0.004	0.024
**Central Unified**	0.0167	0.005	3.398	0.001	0.007	0.026
**Fresno Unified**	0.0190	0.005	4.055	0.000	0.010	0.028
**Fowler Unified**	-0.0254	0.007	-3.668	0.000	-0.039	-0.012
**Pacific Union Elem**	0.1680	0.070	2.401	0.016	0.031	0.305
**Orange Center Elem**	0.0209	0.008	2.563	0.010	0.005	0.037
**Sanger Unified**	0.0183	0.005	3.387	0.001	0.008	0.029
**Washington Colony Elem**	0.0391	0.024	1.614	0.106	-0.008	0.087
**West Fresno Elem**	0.0195	0.006	3.514	0.000	0.009	0.030
**West Park Elem**	0.0070	0.009	0.793	0.428	-0.010	0.024

**Omnibus:**	284789.136	**Durbin-Watson:**	1.750
**Prob(Omnibus):**	0.000	**Jarque-Bera (JB):**	8945615.747
**Skew:**	5.079	**Prob(JB):**	0.00
**Kurtosis:**	28.138	**Cond. No.**	454.

Table S9. *A naïve OLS model is built using panel density calculated at a radius of 1000m. The panel density variable has the largest coefficient and is highly significant.*

Section 6. A comparison of feature importances when panel density is averaged over census tract.

Most of the economic, social, and demographic variables are calculated on the census tract level, whereas the panel density surrounding an address is calculated within variable radii around an address. Therefore, the granularity of these tract-level variables is coarser than that of the density variable. To test for the effect of this granularity difference, we run the model using panel density averaged over the census tract, and find density is still the most important feature. The average tract size is 2.07 square miles.

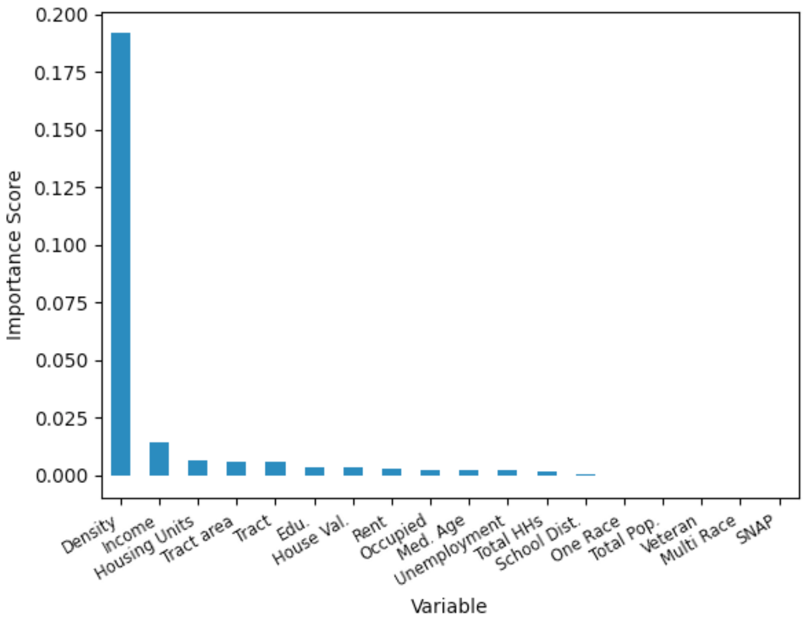

Figure S8. *Feature importances scores are provided for the model when panel density is averaged over census tract.*

We build separate models using tract-averaged density variables calculated at all radii. The following figure shows the feature importance score for each of these tract-averaged density variables, averaged over 50 permutations. We see they have very close importance

scores, as would be expected, given the difference in these variables is on the margins of each tract area.

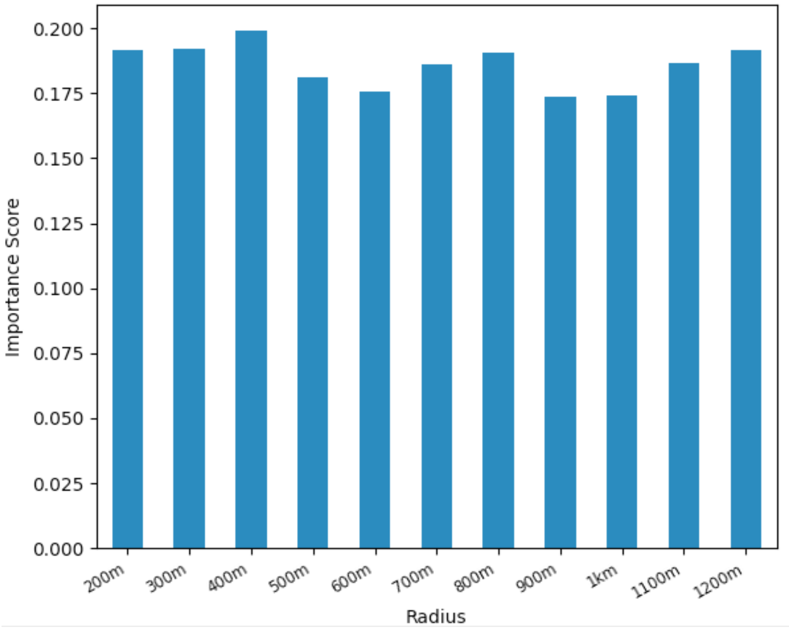

Figure S9. *The feature importance score for each of the tract-averaged density variables.*

Section 7. An evaluation of feature importances without the subtraction of the previous radius.

The following figure provides the importance score for all density variables (200m to 1200m) for the AdaBoost model estimated. For these variables, the normalized panel density at the previous radius is not subtracted.

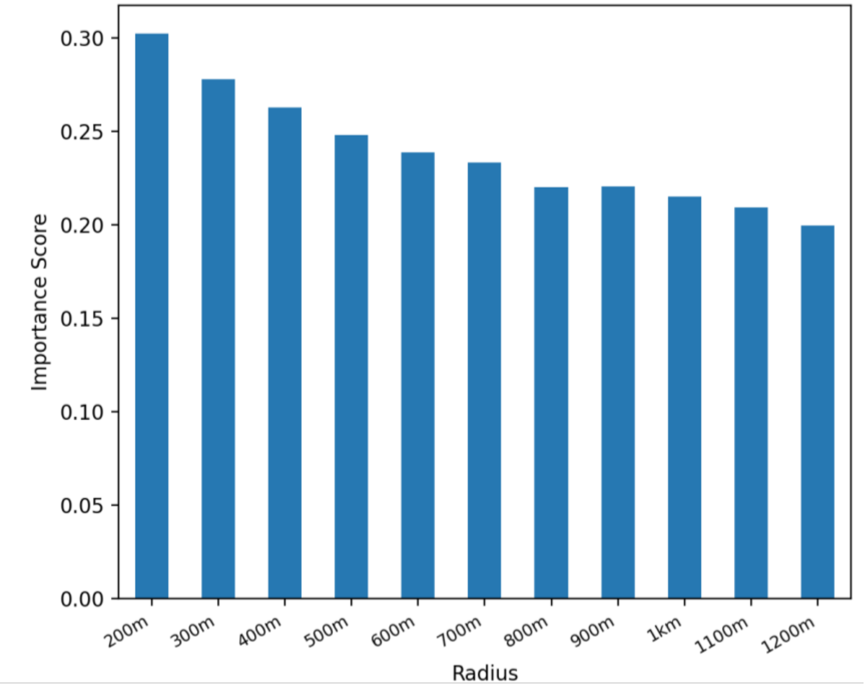

Figure S10. *The feature importance scores for density variables calculated at various radii where the normalized panel density at the previous radius has not been subtracted.*

Section 8. An analysis of the decay in panel density importance over increasing radii when the data is subset by the number of households in the census tract and tract area.

We further explore if the relationship we observe between feature importance and panel density calculation radius is motivated by the difference in data granularity between the density and socioeconomic variables. To this end, we subdivide our data by both the number of households in each tract and tract area (Supplementary Figure S11). We first calculate the feature importances for each of three bins based on the total number of households in the tract, defining a "small" tract as having 1,000 households or less (panel a), "medium" as having between 1,000 and 1,800 households (panel b), and "large" as having more than 1,800 households (panel c). Supplementary Figure S12 shows the exponential decay curve we find for the overall dataset still provides a good representation of the decay of feature importance with an increase in density calculation radius across these subsets.

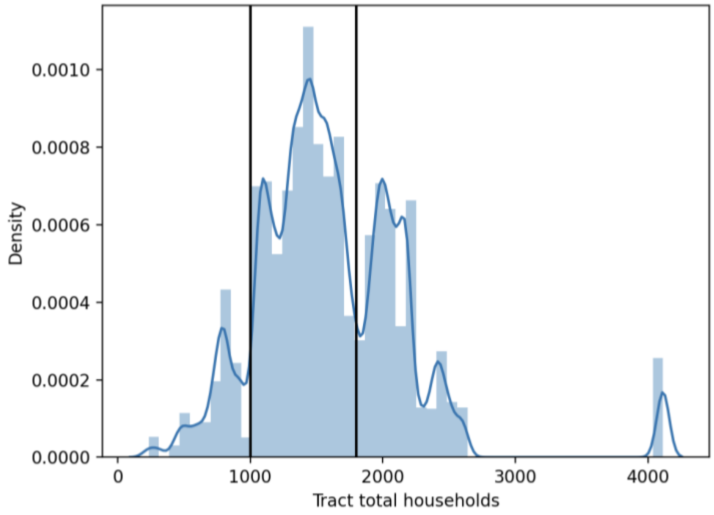

Figure S11. *Distribution of the total households variable (available on the tract level) for all census tracts.*

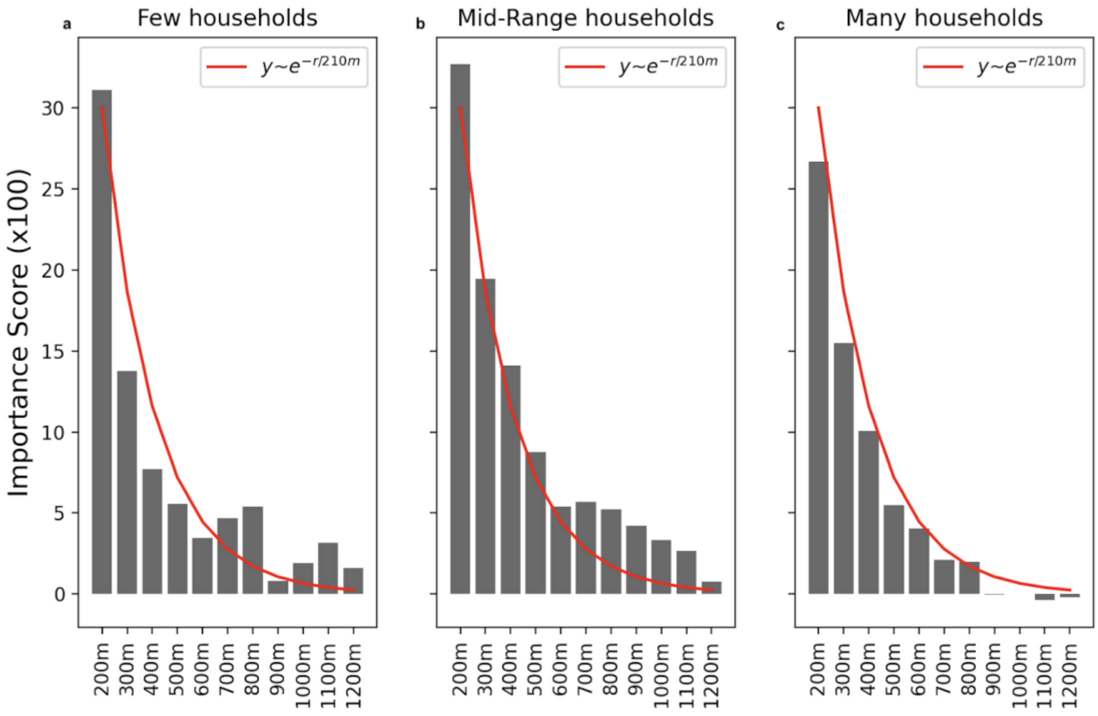

Figure S12. *Feature importance scores, multiplied by 100, for each of the panel density variables calculated for models in which the data is split by census tracts with less than 1,000 total households, between 1,000 and 1,800 total households, and more than 1,800 total households.*

Second, we bin our data over the area of the census tract (Supplementary Figure S13). We define the "small" bin as having an area less than or equal to 1 square mile (panel a), "medium" as having between 1 and 3 square miles (panel b), and "large" as above 3 square miles (panel c). As can be seen from Supplementary Figure S14, the exponential decay curve with a radius of 210m we found for the overall dataset is still a very good fit for the sub-selection of data even through the clarity of the signal is reduced in the smaller data sets, in particular for the "large" area grouping. This is likely due to the overall low numbers of panels (and therefore positive cases in the training data) in this subset.

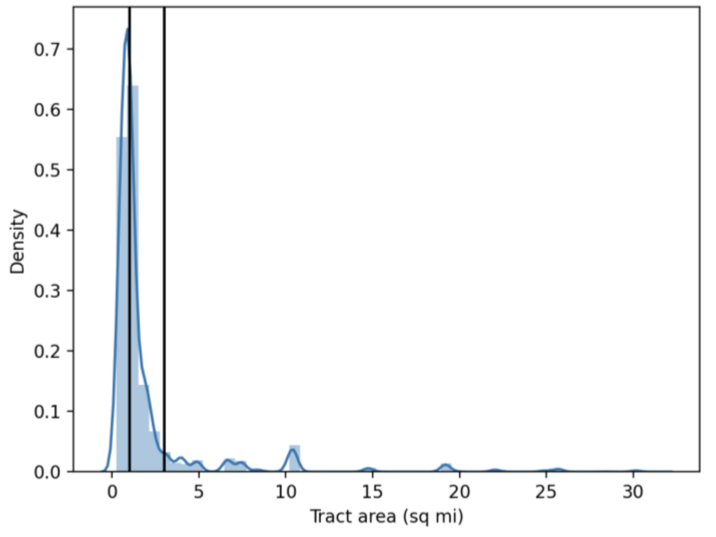

Figure S13. *Distribution of the tract area variable for all census tracts.*

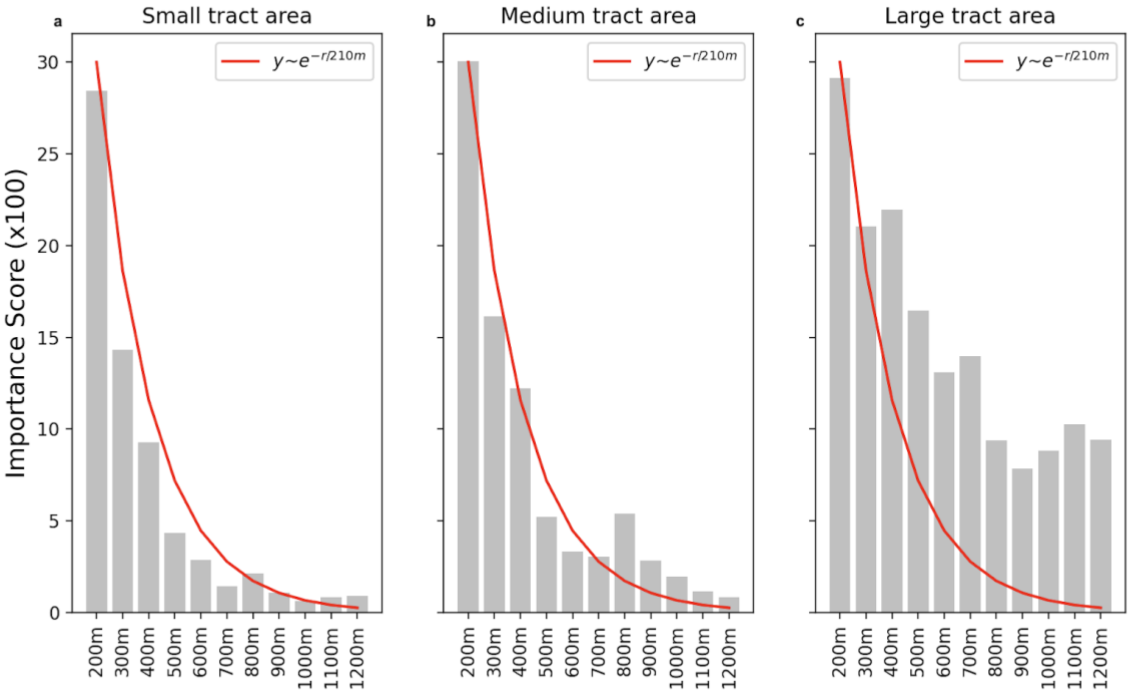

Figure S14. *Feature importance scores, multiplied by 100, for each panel density variable calculated for models in which the data is split by census tracts with a total area less than 1 square mile, between 1 and 3 square miles, and more than 3 square miles.*

Section 9. Presentation of calculated p-values for variables calculated in Figure 3.

P-Values for Figure 3

Density Model	Feature Importance Score (x100)	P-Value
200m	30.7426	3.759971e-92
300m	18.8312	2.326594e-78
400m	14.1525	2.178581e-74
500m	7.7391	1.662433e-70
600m	5.1845	2.776742e-65
700m	4.1772	4.743967e-63
800m	4.6162	6.988895e-61
900m	3.4269	1.858602e-56
1km	1.6573	1.419688e-47
1100m	1.6393	1.218141e-44
1200m	0.8634	4.390670e-35

Table S10. *P-values relevant to the variables included in Figure 3.*

Section 10. Distribution of household income in the dataset, by which the data is subset.

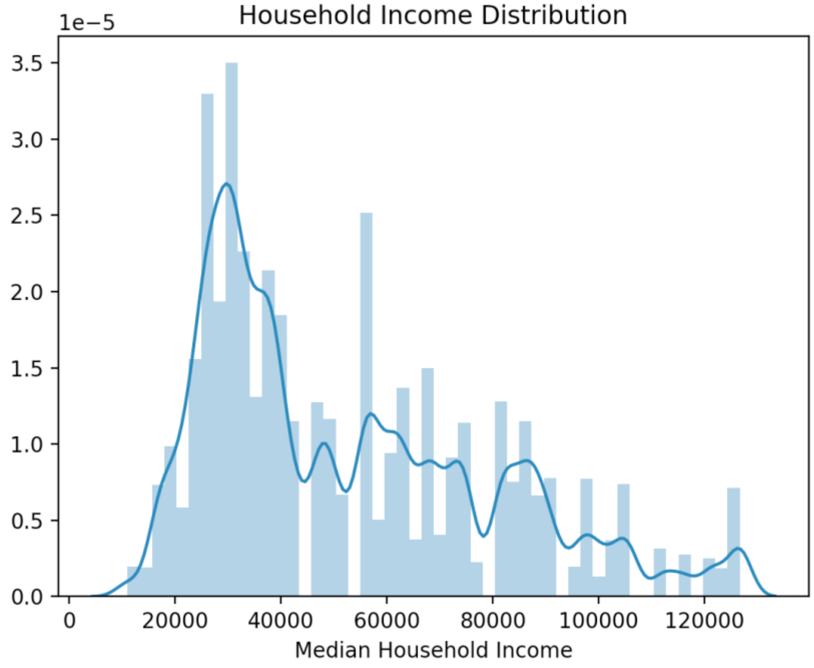

Figure S15. *Distribution of the median household income variable over all census tracts.*

Section 11. Analysis of normalized panel density importance when data is subgrouped by median home value.

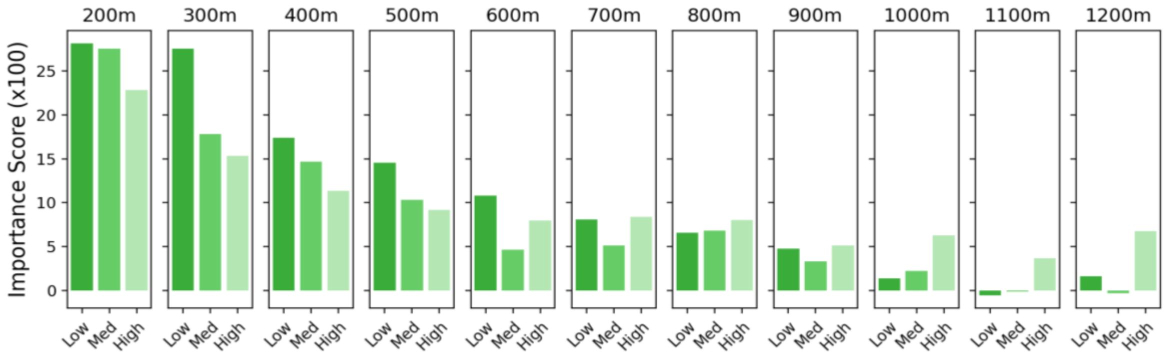

Figure S16. *Feature importance of the normalized panel density variable when the dataset is split into low, medium, and high median home value groups. Low median home value is defined as less than $150,000, medium between $150,000 and $250,000, and high above $250,000. These groupings are based on the variable's distribution.*

Section 12. Analysis of normalized panel density importance when data is subgrouped by income.

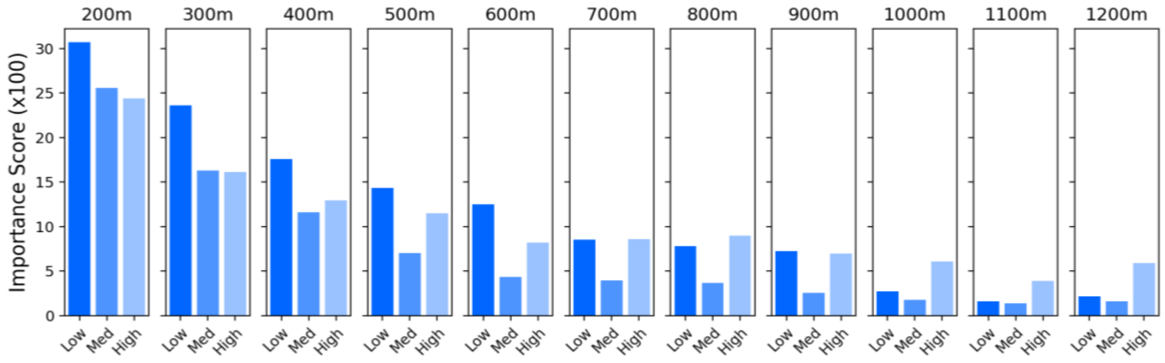

Figure S17. *Feature importance of the normalized panel density variable when the dataset is split into low, medium, and high income brackets.*

Section 13. Descriptive statistics for census tract level features.

Descriptive Statistic for Census Tract Level Features

Name	Mean	Std. Dev.
Income	52659.6274	27340.7914
Med. H. Val	190015.4119	86877.2512
Med. Rent	1059.8113	334.7043
Unemployed	14.7695	6.8003
Tract area	2.0744	4.9092
Single race	0.9219	0.0432
SNAP	269.8316	201.8549
Education	22.7226	15.2776
Total pop	4926.2549	1772.6852
Total HH	1608.1212	582.7259
Veteran	7.1157	2.9588
Occupied	0.9549	0.0266

Table S11. *Mean and standard deviation for those features included in the analysis derived from the American Community Survey, available on the census tract level.*

Section 14. Presentation of the correlation score and direction for all normalized panel density variables with the outcome.

Correlation Score and Direction

Density Model	Correlation	Direction
200m	0.267369	+
300m	0.258520	+
400m	0.240089	+
500m	0.227019	+
600m	0.217599	+
700m	0.214162	+
800m	0.207448	+
900m	0.200474	+
1km	0.193508	+
1100m	0.187575	+
1200m	0.181841	+

Table S12. *Correlation estimate with the outcome (if an address has a panel) and its direction for the normalized density variables at successively increasing radii.*
